# GAS5, a long noncoding RNA, contributes to annulus fibroblast osteogenic differentiation and apoptosis in intervertebral disk degeneration via the miR-221-3p/SOX11 axis

**DOI:** 10.18632/aging.205567

**Published:** 2024-02-23

**Authors:** Qi Liu, Jiaying Luo, Huan Wang, Lei Zhang, Jingwen Guo, Guoxin Jin

**Affiliations:** 1 Department of Orthopedics, Shengjing Hospital of China Medical University, Shenyang 110000, China; 2 School of Life Sciences and Biopharmaceuticals, Shenyang Pharmaceutical University, Shenyang 110000, China; 3 Institute of Health Sciences, China Medical University, Shenyang 110000, China

**Keywords:** annulus fibrosus cells, intervertebral disk degeneration, lncRNA GAS5, miR-221-3p, osteogenesis differentiation, SOX11

## Abstract

miR-221-3p has been reported to attenuate the osteogenic differentiation of annulus fibrosus cells (AFs), which has been implicated in intervertebral disk degeneration (IVDD) development. This study aimed to elucidate miR-221-3p’s role in osteogenic differentiation and apoptosis of AFs in an IVDD model. After successfully establishing an IVDD rat model by annulus fibrosus needle puncture, AFs were isolated. Bioinformatics, dual-luciferase reporter, and AGO2-RNA immunoprecipitation (RIP) assays predicted and confirmed the potential miR-221-3p lncRNA and gene target. Functional analyses were performed after AF transfection to explore the roles of the identified lncRNA and gene. Western blotting, Alkaline phosphatase (ALP), and Alizarin red and TUNEL staining were performed to investigate AF apoptosis and osteogenic differentiation with different transfections. Compared with AFs isolated from sham rats, IVDD-isolated Afs exhibited stronger osteogenic potential and higher apoptosis rates accompanied by miR-221-3p downregulation. The growth arrest-specific transcript 5 (GAS5) was identified as miR-221-3p’s target lncRNA, which was highly expressed in IVDD. GAS5 overexpression facilitated AF apoptosis and osteogenic differentiation, whereas silencing GAS5 had the opposite effect. SRY box-related11 (SOX11) was identified as a downstream miR-221-3p target gene in IVDD. GASS silencing-induced suppression of AF apoptosis and osteogenic differentiation could be reversed by SOX11 overexpression. Our findings uncovered a lncRNA GAS5/miR-221-3p/SOX11 axis in Afs under IVDD, which may help implement novel IVDD therapeutic strategies.

## INTRODUCTION

Lower back pain (LBP), as one of the most common causes of activity limitations and neurological deficit, is responsible for roughly 70–85% of chronic pain cases [1]. Intervertebral disk degeneration (IVDD) is the predominant LBP trigger, accounting for 40% of all LBP-inducing factors [2, 3]. Typically, IVDD can be alleviated by various drugs, including opioid analgesics [4] and nonsteroidal anti-inflammatory drugs [5]. However, the specific pathological mechanism underlying IVDD remains elusive.

Anatomically, the intervertebral disk (IVD) is characterized as fibrocartilaginous [6] and mainly consists of three component constructs, including gelatinous proteoglycan-rich nucleus pulposus (NP) in the center, annulus fibrosus (AF) in the surrounding area, and cartilaginous lower and upper endplates [7]. NP cell collapse and NP characteristic loss leads to decreased AF mechanical resistance with consequent degradation of the entire IVD [8]. Although multiple cell types differences are observed during the late stages of IVDD, such as osteoblasts and neurons, the mechanism triggering these changes is still unclear [9]. An increasing number of studies support the idea that IVD contains cells capable of differentiation. For instance, Jin et al. were the first to demonstrate that AF cells could differentiate into chondrocytes and osteoblasts *in vitro* and *in vivo* [10]. Moreover, the authors revealed that miR-221-3p suppresses the osteogenic differentiation of degenerated AFs [11]. It has also been reported that miR-221-3p can suppress cell apoptosis in several diseases [12, 13], and excessive AF apoptosis is highly associated with IVDD progression [14]. Therefore, miR-221-3p may be a potent regulator that protects against IVDD. However, the underlying role of miR-221-3p in IVDD has yet to be investigated.

The involvement of noncoding RNAs (ncRNAs), such as long noncoding RNAs (lncRNAs) and microRNAs (miRNAs), in the pathogenesis and progression of various diseases, including IVDD, has already been confirmed. MiRNAs can guide the post-transcriptional repression of protein-coding genes containing specific miRNA response elements [15]. The lncRNAs can harbor a miRNA response element to compete with other RNA transcripts, indirectly rendering other RNA(s) free from miRNA regulation [16]. Such indirect interactions are regulated by competing endogenous RNA (ceRNA) regulatory networks, which play a critical role in IVDD development [17, 18]. Jiang et al. demonstrated that lncRNA FAM83H-AS1 sponged miR-22-3p to regulate NP cell growth and IVDD homeostasis [19]. In addition, LncRNA NEAT1 silence inhibited BAX/BAK pathway activity to attenuate IVDD through miR-195a upregulation [20]. Therefore, miR-221-3p may play a role in the apoptosis and osteogenic differentiation of AFs with a distinct involvement of its target lncRNA and mRNA in IVDD.

Based on the findings above, the present study aimed to identify the underlying ceRNA network of miR-221-3p in IVDD, providing valuable scientific information to develop novel therapeutic strategies for patients with IVDD.

## RESULTS

### Characterization of IVDD rat model-derived AFs

**Figure 1:**
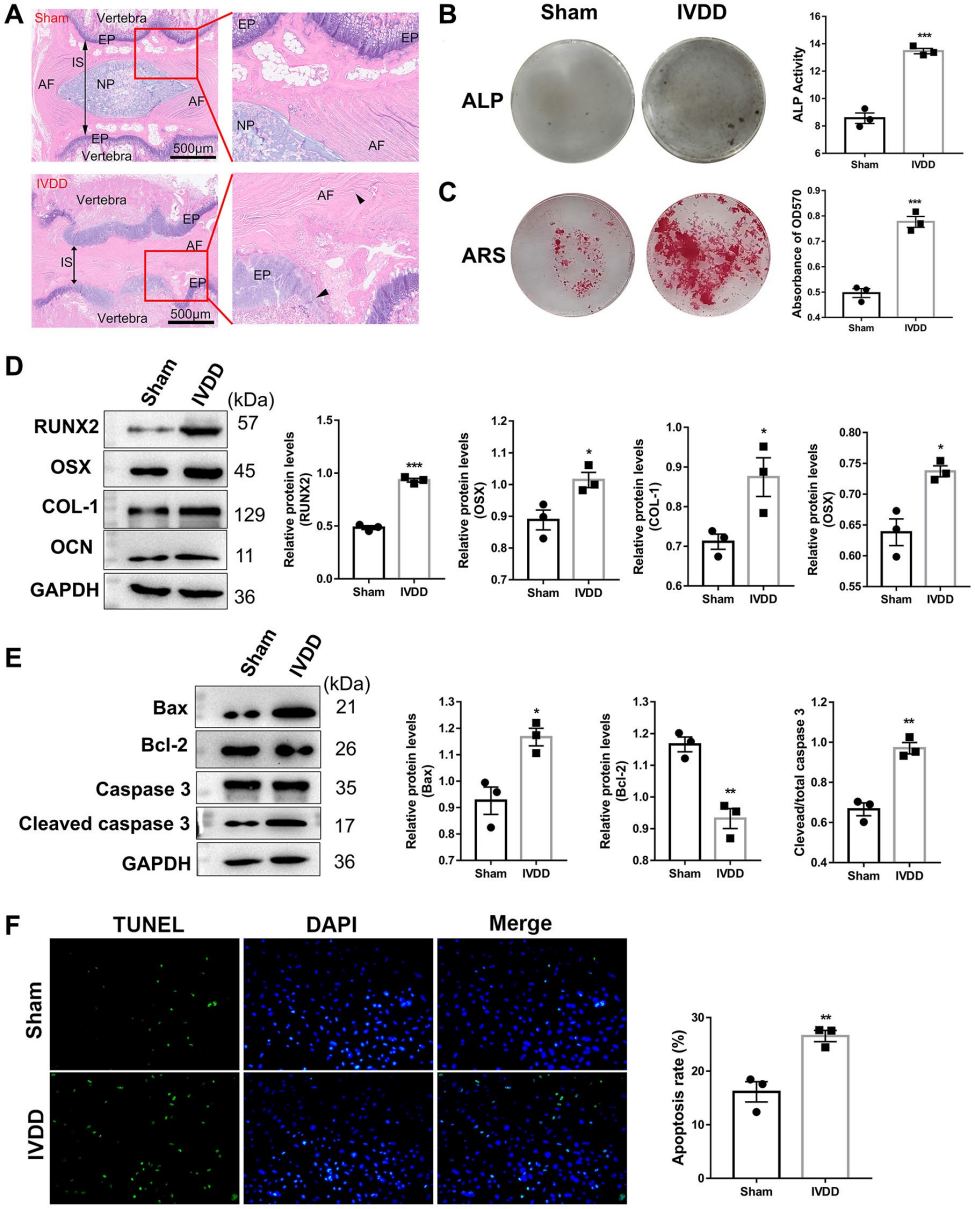
**Characterization of IVDD rat model-derived Afs.** (**A**) HE staining of Co6-7 intervertebral disk of rats from the sham and IVDD groups (*n* = 6). Abbreviations: AF: annulus fibrosus; NP: nucleus pulposus; IS: intervertebral space; EP: endplate. Scale bar = 500 μm. The black arrows represent AF and EP cells, respectively. (**B**) ALP and (**C**) ARS staining assays were used to evaluate the osteogenic differentiation level (*n* = 3). (**D**) The markers for osteogenic differentiation were detected using a western blotting assay (*n* = 3). (**E**) A western blotting assay examined apoptosis-related protein levels. (*n* = 3) (**F**) TUNEL assay was performed to analyze the apoptosis of AFs (*n* = 3). (^*^p < 0.05, ^**^p < 0.01, and ^***^p < 0.001).

We evaluated the histological structure of the IVD after H&E staining to confirm the IVDD rat model was successfully established (Figure 1A). The histological structure of Co6-7 in the IVDD group displayed inflammatory cell infiltration, gelatinous NP structure disorder in AF, and even narrowing of intervertebral space (IS) between endplate (EP) compared with the sham group. Calcification of the IVD tissues was highly correlated with IVDD progression. After successfully isolating AFs from the Co6-7 of the sham and IVDD rat models, the AF ostenogenic differentiation ability was investigated by ALP and Alizarin red staining (ARS) assays. The ALP activity was elevated in IVDD-derived AFs (Figure 1B), and the matrix mineralization level was significantly increased (Figure 1C). In addition, western blotting (WB) analysis demonstrated that osteogenic differentiation-related markers, including RUNX2, OSX, COL-1, and OCN were highly expressed in AFs from IVDD rats compared with those from sham group (Figure 1D). These data suggested that IVDD group AF have stronger potential for differentiating into osteoblasts compared with those of the sham group, which indirectly confirmed that the IVDD rat model was successfully established. Moreover, compared with the sham group, the expression levels of pro-apoptosis proteins (bax and cleaved caspase-3) of AFs were considerably elevated in the IVDD group, as opposed to the anti-apoptosis protein, bcl-2, which was significantly reduced (Figure 1E). This indicated that IVDD AF cells underwent excessive apoptosis, which was confirmed by TUNEL staining (Figure 1F). Taken together, these data demonstrated that AFs were successfully isolated from the sham and IVDD rat models.

### lncRNA GAS5 is highly expressed in IVDD-derived AFs and sponges miR-221-3p

**Figure 2:**
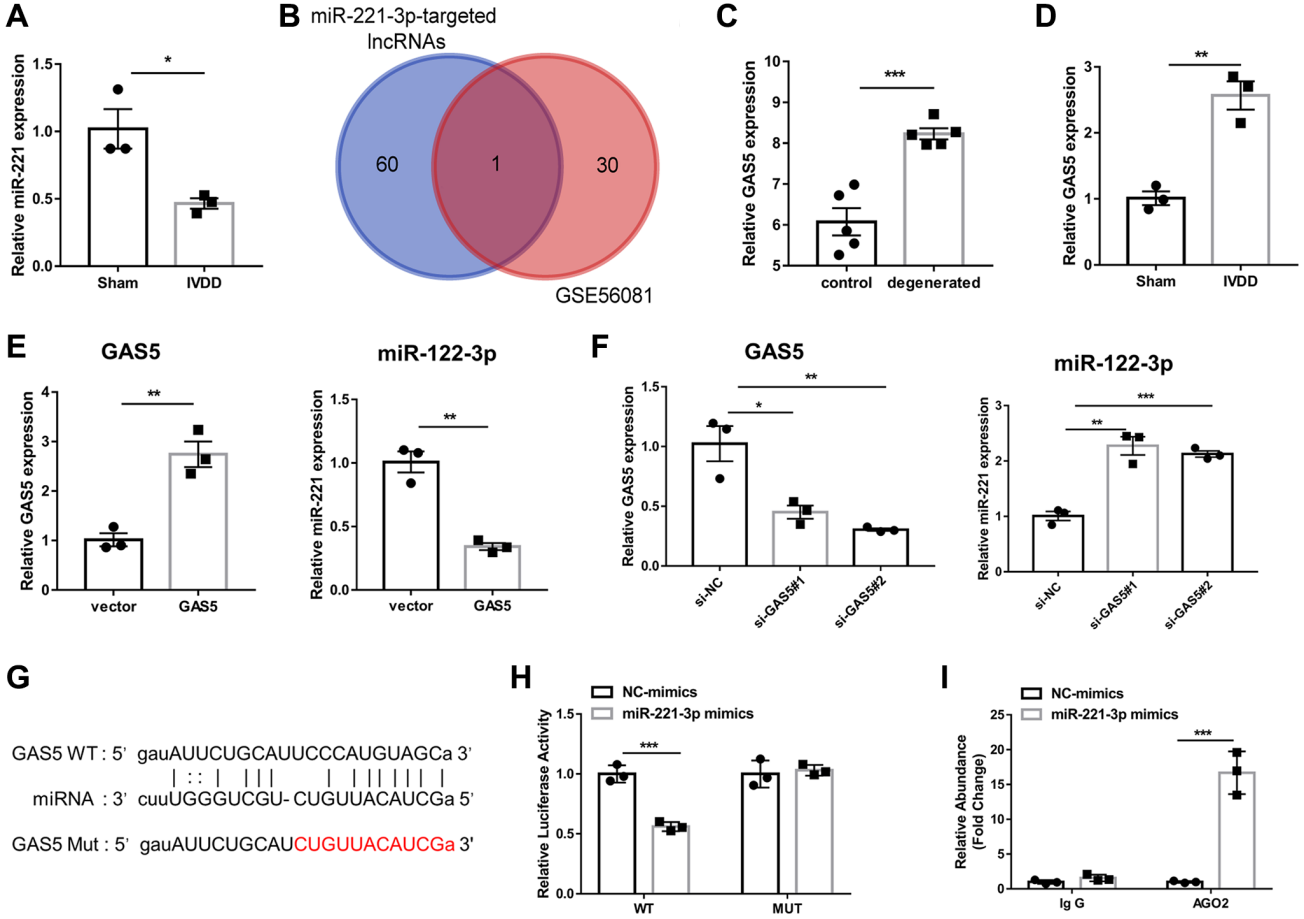
**LncRNA GAS5 is upregulated in IVDD-derived AFs and inhibits miR-221-3p expression.** (**A**) qRT-PCR was adopted to examine the expression of miR-221-3p in AFs. (*n* = 6) (**B**) The Venn diagram was drawn to obtain the common lncRNA of upregulated lncRNA in IVDD based on the GSE56081 dataset, and ENCORI predicted the potential target lncRNA of miR-221-3p. (**C**) The expression level of GAS5 between normal and degenerative disks on the GSE56081 dataset. (*n* = 5) (**D**) qRT-PCR assay was utilized to detect the expression of GAS5 in AFs isolated from sham and IVDD rats (*n* = 3). QRT-PCR detected (**E**) The expression levels of GAS5 and miR-221-3p in Afs after transfection with GAS5 overexpression plasmid and an empty vector. (*n* = 3) (**F**) The expression levels of GAS5 and miR-221-3p in Afs were detected by qRT-PCR after transfection with si-GAS5 and si-NC (*n* = 3). (**G**) The potential binding sites between GAS5 and miR-221-3p predicted by ENCORI (*n* = 3). (**H**) Luciferase activity and (**I**) AGO2-RIP were employed to evaluate the binding between lncRNA GAS5 and miR-221-3p (*n* = 3). (^*^p < 0.05, ^**^p < 0.01, and ^***^p < 0.001).

MiR-221-3p has been proven to be lowly expressed in IVDD-derived AFs [11], which was consistent with the results of the present study (Figure 2A). To obtain the potential target lncRNA of miR-221-3p in IVDD, we identified 31 upregulated lncRNA in IVDD based on the GSE56081 dataset (Supplementary Table 1). We intersected them with 61 potential target lncRNAs of miR-221-3p predicted by ENCORI (Supplementary Table 2). The Venn diagram displayed that only a common lncRNA, GAS5, was obtained after intersection (Figure 2B). In the GSE56081 dataset, GAS5 expression in degenerated disks was significantly higher than in normal disks (Figure 2C). Similarly, GAS5 expression of AFs from the IVDD group was also significantly higher than that in the sham group (Figure 2D). Consequently, GAS5 overexpression was found to significantly inhibit miR-221-3p expression of AFs (Figure 2E). At the same time, silencing of GAS5 could promote the expression of miR-221-3p in AFs (Figure 2F). After predicting the potential binding site between GAS5 and miR-221-3p (Figure 2G), dual-luciferase reporter assay revealed that miR-221-3p mimic considerably reduced the luciferase activity of a reporter gene with wild-type (WT). This finding was not observed with mutant (MUT) GAS5 3′-UTR (Figure 2H). AGO2-RIP assay further demonstrated that GAS5 was efficiently enriched in anti-AGO2 complexes in AFs (Figure 2I). These findings suggested that lncRNA GAS5 was highly expressed in IVDD and could downregulate miR-221-3p expression in AF cells.

### lncRNA GAS5 promotes AFs apoptosis and osteogenic differentiation by miR-221-3p

**Figure 3:**
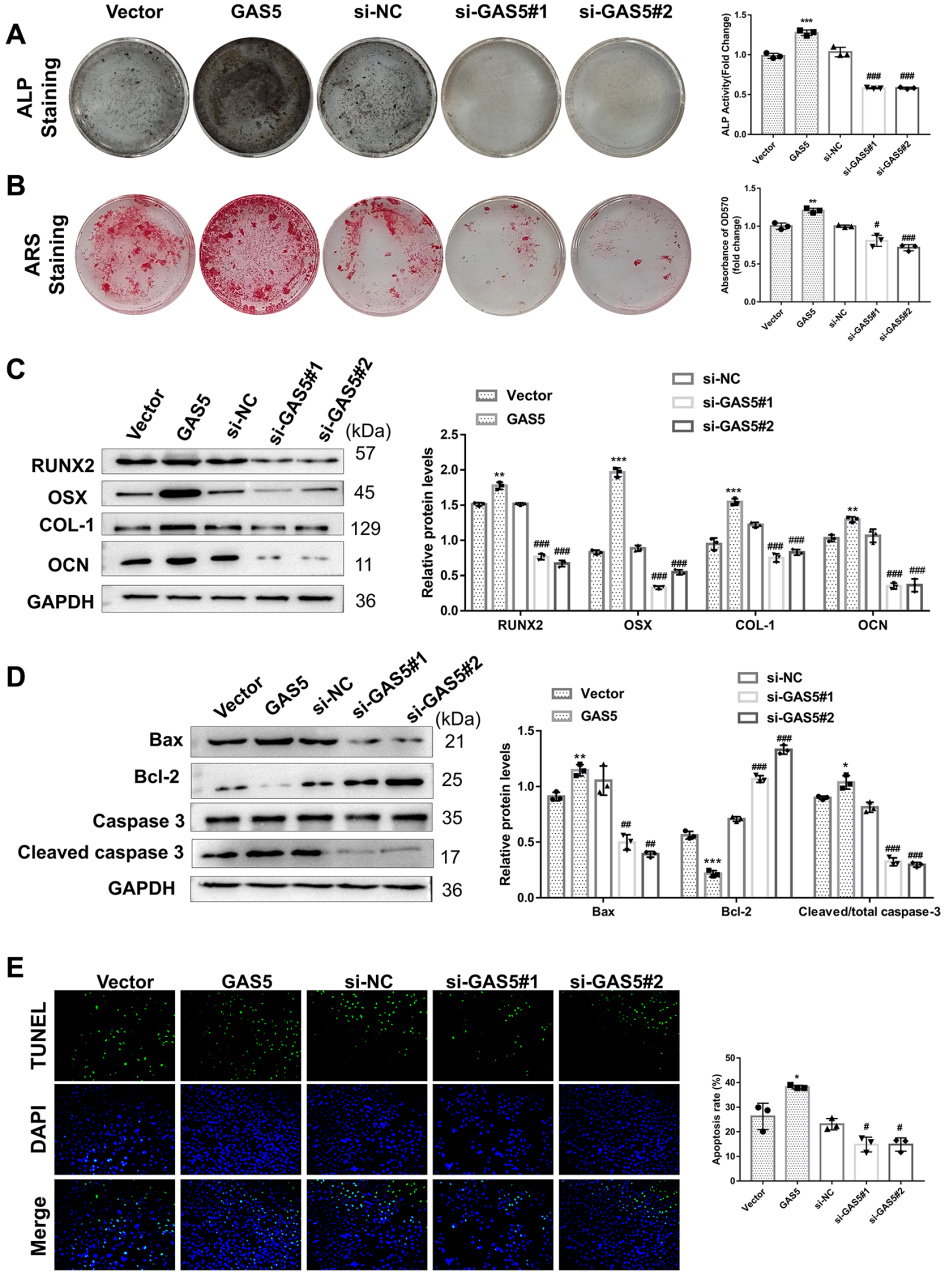
**LncRNA GAS5 facilitates Afs apoptosis and osteogenic differentiation.** AFs were transiently infected with the overexpression plasmid or GAS5 siRNA, while an empty vector and si-NC were used as negative controls. (**A**) The osteogenic differentiation-related markers were detected using a western blotting assay (*n* = 3). The osteogenic differentiation level of AFs with different transfections was evaluated by (**B**) ALP and (**C**) ARS assays. (*n* = 3) (**D**) The expressions of apoptosis-related proteins were detected by western blotting (*n* = 3). (**E**) TUNEL staining assay was performed to analyze the apoptosis of AFs (*n* = 3). (^**^p < 0.01 and ^***^p < 0.001, vs. the vector group; ^#^*p* < 0.05, ^##^*p* < 0.01, and ^###^*p* < 0.001, vs. the si-NC group).

To evaluate the functional effect of GAS5 on AFs, GAS5 was overexpressed or knocked down in AFs. Although GAS5 overexpression significantly promoted the expression of osteogenic differentiation-related proteins (RUNX2, OSX, COL-1, and OCN) in AFs, the opposite effect was observed fromGAS5 silencing (Figure 3A). Upon osteogenic induction, GAS5 overexpression significantly increased tALP activity (Figure 3B) and AF matrix mineralization (Figure 3C), which were reduced when silencing GAS5. These results collectively revealed the promotive role of GAS5 in AF osteogenic differentiation. Meanwhile, GAS5 overexpression led to a significant increase in the expression of pro-apoptosis proteins and a decrease in the anti-apoptosis protein expression in AFs (Figure 3D), suggesting that GAS5 contributed to the apoptosis of AFs. The TUNEL staining assay exhibited a similar tendency, showing that the number of TUNEL-positive AFs was significantly increased after overexpressing GAS5 and decreased after knocking down GAS5 (Figure 3E). The above findings indicated that GAS5 contributed significantly to osteogenic differentiation and apoptosis in AFs.

Moreover, we verified whether the regulation of GAS5 on the apoptosis and osteogenic differentiation of AFs relied on miR-221-3p (Supplementary Figure 1). The results showed that the promotive role of GAS5 in the osteogenic differentiation (Supplementary Figure 1A–1C) and apoptosis (Supplementary Figure 1D, 1E) of AFs was blocked after overexpressing miR-221-3p. GAS5 is involved in AF apoptosis and osteogenic differentiation by regulating miR-221-3p.

### miR-221-3p negatively regulates SOX11 expression in Afs

**Figure 4:**
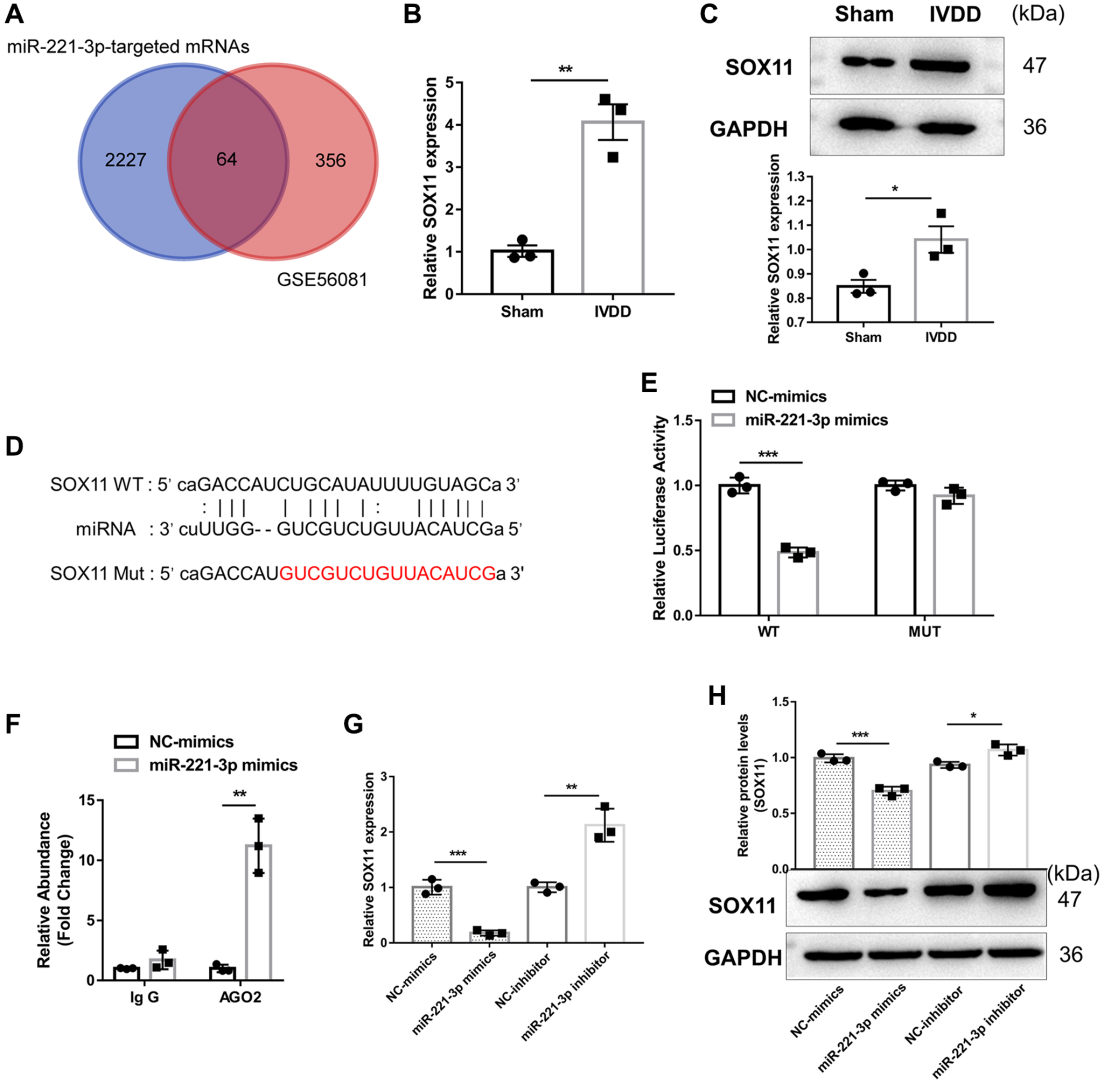
**miR-221-3p suppresses SOX11 expression.** (**A**) The Venn diagram was drawn to obtain the common lncRNA of upregulated mRNA in IVDD based on the GSE56081 dataset and the potential target mRNA of miR-221-3p predicted by ENCORI. (**B**) qRT-PCR and (**C**) western blotting were utilized to detect mRNA and protein expression of SOX11 in AFs isolated from sham and IVDD rats, respectively (*n* = 3). (**D**) The potential binding sites between GAS5 and miR-221-3p predicted by ENCORI. (**E**) Luciferase activity and (**F**) AGO2-RIP were employed to evaluate the binding between lncRNA GAS5 and miR-221-3p (*n* = 3). (**G**) qRT-PCR and (**H**) western blotting were used to examine the expression levels of SOX11 in AFs with miR-221-3p overexpression or silencing (*n* = 3). (^*^p < 0.05, ^**^p < 0.01, and ^***^p < 0.001).

Then, we explored the downstream RNA of miR-221-3p to uncover its ceRNA axis further in the osteogenic differentiation and apoptosis in AFs. To obtain the potential target mRNA of miR-221-3p in IVDD, we identified 420 upregulated mRNAs in IVDD based on the GSE56081 dataset (Supplementary Table 3) and merged them with 2291 potential target mRNAs of miR-221-3p predicted by ENCORI (Supplementary Table 4). Sixty-four common mRNAs were obtained after intersection (Supplementary Table 5) (Figure 4A). Since the osteogenic differentiation potential was highly correlated with AF stemness, SOX11, a mesenchymal stem cell characteristic gene [21], was selected. Our findings showed that SOX11 was highly expressed by mRNA and protein in IVDD-derived Afs (Figure 4B, 4C). ENCROEI predicted the binding sites between miR-221-3p, and SOX11 (Figure 4D). Dual-luciferase reporter assay illustrated that miR-221-3p decreased luciferase activity in SOX11-WT plasmid transfected cells (*p* < 0.01), as opposed to no change observed in cells transfected with SOX11-MUT plasmid (Figure 4E), confirming a direct interaction between miR-221-3p and SOX11. The AGO2-RIP assay demonstrated that miR-221-3p was efficiently enriched in anti-AGO2 complexes in Afs compared with the anti-IgG group (Figure 4F). Furthermore, miR-221-3p overexpression could significantly suppress SOX11 expression. In contrast, the opposite effect was noted when silencing miR-221-3p (Figure 4G, 4H). Further, the biological experiments revealed that miR-221-3p overexpression could significantly suppress the AF osteogenic differentiation (Supplementary Figure 2A–2C) and apoptosis (Supplementary Figure 2D, 2E), while these effects were rescued by SOX11 overexpression. MiR-221-3p could directly inhibit SOX11 expression to regulate apoptosis and osteogenic differentiation in AFs.

### lncRNA GAS5 promotes apoptosis and osteogenesis differentiation of AFs via the miR-221-3p/SOX11 axis

**Figure 5:**
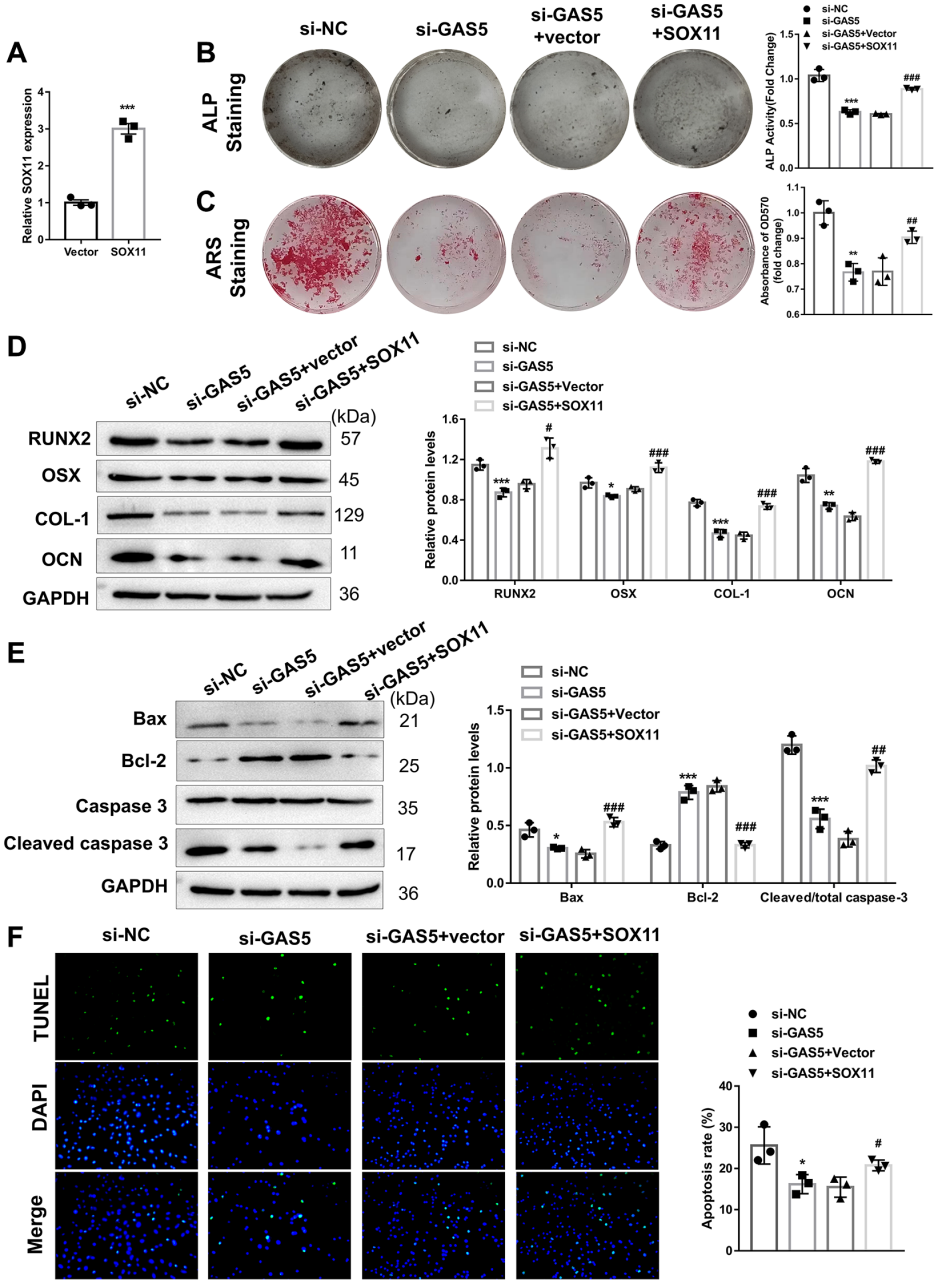
**The role of GAS5 in the apoptosis and osteogenic differentiation of AFs was mediated by SOX11.** (**A**) AFs were transfected with SOX11 overexpression or an empty vector, and the transfection efficacy was detected using qRT-PCR (*n* = 3). Next, AFs were transfected with or without si-GAS5 plasmid alone or with the SOX11 overexpression vector. (**B**) ALP and (**C**) ARS staining assays were used to evaluate the osteogenic differentiation level (*n* = 3). (**D**) The osteogenic differentiation-related markers were detected using western blotting (*n* = 3). (**E**) The apoptosis-related protein levels were measured using western blotting (*n* = 3). (**F**) TUNEL staining assay was employed to evaluate the apoptosis of AFs (*n* = 3). (^*^p < 0.05, ^**^p < 0.01, and ^***^p < 0.001, vs. the vector group; ^#^*p* < 0.05, ^##^*p* < 0.01, and ^###^*p* < 0.001, vs. the si-GAS5 group).

To explore whether GAS5 regulates the apoptosis and osteogenic differentiation of AFs in a SOX11-dependent manner, AFs were transfected with or without si-GAS5 plasmid (either alone or with SOX11 overexpression plasmid). Our results showed that the SOX11 overexpression plasmid could successfully overexpress SOX11 in AFs (Figure 5A). As expected, the previously inhibited ALP activity (Figure 5B) and matrix mineralization level (Figure 5C) by si-GAS5 transfection were partly rescued by SOX11 overexpression in AFs, indicating that SOX11 mediated the role of GAS5 in the osteogenic differentiation of AFs. In addition, suppression of osteogenic differentiation-related proteins (RUNX2, OSX, COL-1, and OCN) in GAS silencing AFs was significantly restored by SOX11 overexpression (Figure 5D). Concerning the apoptosis of AFs, SOX11 overexpression also restrained the effect of GAS silencing, as evidenced by the WB and TUNEL assay. SOX11 overexpression in GAS5-silenced AFs was found to elevate pro-apoptosis proteins’ expression levels, decrease anti-apoptosis proteins’ expression, and increase TUNEL-positive cells (Figure 5E, 5F). These findings suggested that GAS5 contributes to the apoptosis and osteogenic differentiation of AFs in IVDD via the miR-221-3p/SOX11 axis.

## DISCUSSION

The present study identified GAS5 as a ceRNA of miR-221-3p, which was highly expressed in IVDD-derived AFs and contributed to the apoptosis and osteogenic differentiation of AFs in IVDD. lncRNA GAS5 promoted AF cell apoptosis and osteogenic differentiation. Moreover, we confirmed that SOX11 is a downstream regulator of GAS5/miR-221-3p in the apoptosis and osteogenic differentiation of AFs.

As mentioned above, many research studies have concentrated on the NP cells to alleviate IVDD; however, as an important component in the biomechanical construction of IVDD, the structural integrity of AFs is closely associated with IVDD development. Recently, Jing et al. confirmed that FoxO1a mediated the apoptosis of AFs, which caused IVDD [22]. Furthermore, Xu et al. suggested that PGC-1α mediated Sirtuin2 impedes the apoptosis of AFs induced by oxidative stress by inhibiting cell mitophagy in IVDD [23]. Moreover, it has been confirmed that osteogenic differentiation can occur in AFs in response to stimuli, a process regulated by miR-221-3p [11]. To uncover the regulatory mechanism underlying the effect of miR-221-3p on the osteogenic differentiation and apoptosis of AFs in IVDD, we identified the potential lncRNA and mRNA that target miR-221-3p by using a bioinformatics method based on the GEO and ENCORI databases on the IVDD-isolated AFs.

In this study, only one lncRNA, GAS5, was predicted lncRNA target of miR-221-3p in IVDD. Extensive studies have shown that GAS5 plays an important role in various diseases, such as diabetic wound healing [24], asthma [25], pneumonia [26], and several types of cancers, mainly including cholangiocarcinoma [27], laryngeal squamous cell carcinoma [28], and osteosarcoma [29]. GAS5 has also been proven to contribute to cell apoptosis [25], inflammation [26], and autophagy [30]. GAS5 can promote the osteogenic differentiation of bone marrow mesenchymal stem cells via the miR-135a-5p/FOXO1 axis regulation [31]. We indicated that GAS5 could functionally facilitate apoptosis and osteogenic differentiation of AFs in IVDD, which is consistent with previous reports.

Many lncRNAs exert various functional effects on multiple biological processes by acting as miRNA sponges [32]. GAS5 has also been reported to act as a ceRNA of different miRNAs. For example, GAS5 regulated T Cell function via miR-21-mediated signaling in people with HIV [33]. GAS5 has a role in pneumonia by regulating the microRNA-222-3p/TIMP3 axis [34]. Herein, dual-luciferase reporter and AGO2-RIP assays suggested that GAS5 acted as a sponge of miR-221-3p in AFs. Functional experiments further verified the regulatory role of the GAS5/miR-221-3p axis in the apoptosis and osteogenic differentiation of Afs. It has been reported that SOX11 can promote osteoblast progenitor survival and proliferation, enhancing the differentiation of early osteoblasts [35]. Therefore, SOX11 was selected among 64 identified genes for further analysis. A direct interaction between miR-221-3p and SOX11 was verified, leading us to speculate that the miR-221-3p/SOX11 axis might mediate the effect of GAS on IVDD-isolated AFs. Our functional experiments finally confirmed that the impact of silencing GAS5 on the apoptosis and osteogenic differentiation of AFs could be reversed by SOX11 overexpression.

To summarize, our study demonstrated that GAS5 might be critical for IVDD development, as it might function as a ceRNA to sponge miR-221-3p to upregulate SOX11, promoting AF apoptosis and osteogenic differentiation. These findings enriched our knowledge of the role of AFs in developing IVDD, which may provide a novel insight for subsequent IVDD treatments.

## MATERIALS AND METHODS

### IVDD rat model

All animal experiments complied with the ARRIVE guidelines and were performed in accordance with the UK Animals (Scientific Procedures) Act, 1986 and associated guidelines, EU Directive 2010/63/EU for animal experiments, and the National Institutes of Health Guide for The Care and Use of Laboratory Animals (NIH Publications No. 8023, revised 1978). Furthermore, all animal experiments were approved by the Animal Ethics Committee of the Shengjing Hospital of China Medical University. Twelve Sprague-Dawley (SD) rats (female, three months old) were obtained from Liaoning Changsheng Biotechnology Co., Ltd., (Liaoning, China) to construct the IVDD model. Initially, rats were fed normally, and the IVDD model was established using AF needle puncture [20]. In brief, rats were fasted for 24 hours before the operation and were anesthetized by intraperitoneal injection of 2% pentobarbital sodium (50 mg/kg). Next, to establish the IVDD model (*n* = 6), a 21-gage needle was inserted into the center of the coccygeal disks (Co6-7), which was limited to 5 mm depth to ensure no further damage.

Consequently, the needle was turned twice and kept in place for 30 seconds [36]. All processes were performed similarly for the sham group (*n* = 6), except for the puncture operation. Twenty-eight days after the operation, the rats were sacrificed, and the Co6-7 IVD obtained from the two groups were maintained for further research.

### Hematoxylin-eosin (H&E) staining

H&E staining was performed to confirm the successful establishment of the IVDD model as previously described [37, 38]. Briefly, the IVD was decalcified and fixed in 4% formaldehyde, dehydrated, and then embedded in paraffin. The slides were stained with H&E and then imaged with a microscope.

### Isolation and cell culture of AFs

AFs from both groups were isolated from surgically removed tissue from sham and IVDD model rats. After successfully constructing the IVDD rat model, the disks were cut from the osseous endplate using a sharp Cobb elevator, then the outermost AF layer and the inner AF, closing the NP. Next, the remaining AF-containing area was dissected into small pieces and digested using collagenase (0.01%, Crescent Chemical, NY, USA) for 3 h at 37°C and centrifuged to remove the supernatant. The remaining cell pellet was added into DMEM (Dulbecco’s Modified Eagle Medium) containing erythrocyte lysis buffer (160 mM of NH_4_Cl) and cultured for 10 min with gentle agitation. Then, AFs were added to DMEM/F12 medium with 10% fetal bovine serum (Life Technology, Carlsbad, CA, USA) and maintained at 37°C with 5% CO_2_. AFs at 2–4 passages were then used for further investigations.

### Osteogenesis differentiation induction

For osteogenic differentiation, the AFs from each group were incubated in osteogenic induction media in a 24-well plate until 85% confluency. The constitutes of the osteogenic induction media mainly consisted of DMEM/F12, according to previous reports [11].

### Cell transfection

The pcDNA-GAS5, pcDNA-SOX11, miR-221-3p-mimics, lncRNA GAS5 siRNAs, and SOX11 siRNAs were purchased and designed by GeneChem Corporation (Shanghai, China) (Table 1). AFs were transfected with indicated plasmids by lipofectamine (Vision 2000, 11668-019, Invitrogen, USA) and cultured for 48 h for further research.

**Table 1:** shRNA sequences against specific targets.

si-GAS5-1	5′–3′	GAUGGAGUCUCAUGGCACA
si-GAS5-2	5′–3′	UGGAUGACUUGCUUGGGUA
si-SOX11	55′–3′	GCCTCTACTACAGCTTCAAGAAC

### Alkaline phosphatase (ALP) staining and enzyme activity assay

The ALP assay kit (Beyotime, Shanghai, China) was used to evaluate the AF osteogenic differentiation in accordance with the manufacturer’s instructions, and the images were observed and captured using light microscopy (Nikon TS100, Japan). The SensoLyte^®^ pNPP Alkaline Phosphatase Assay Kit (AnaSpec, Fremont, CA, USA) was adopted for ALP activity analysis. In brief, cell lysates were added into p-nitrophenyl phosphate (pNPP) and mixed for 30 min, and then the OD values were examined at 570 nm.

### Alizarin red staining (ARS)

ARS was performed to evaluate cell mineralization. Briefly, Alizarin Red S reagent (#ST1078-25 g; Beyotime Biotech) was used to stain the cells for 5 min at 37°C, according to the manufacturer’s instructions. The images were obtained using a microscope (Olympus, Tokyo, Japan), and quantification was performed with ImageJ software.

### TUNEL assay

For AFs apoptosis analysis, the TUNEL staining kit (Beyotime, Shanghai, China) was used per the manufacturer’s protocols. Images were obtained using a microscope (Olympus, Tokyo, Japan) under randomly selected fields to calculate TUNEL-positive cell numbers. The red staining in the nucleus indicated the apoptosis level of AFs.

### Western blotting (WB)

Total protein was obtained from AFs using lysis buffer (Beyotime, China) and separated using SDS-PAGE gels (Jinsirui, Nanjing, China). The protein samples were transferred onto PVDF membranes (Merck Millipore, Burlington, MA, USA), incubated in PBS containing 10% skim milk for 1 h, and mixed with primary antibodies at 4°C overnight. The blots were then incubated with a secondary antibody conjugated with horseradish peroxidase (HRP) (Cell Signaling Technology, Danvers, MA, USA). The protein expression levels were analyzed using ImageJ software (National Institutes of Health, Bethesda, MD, USA). GAPDH (ab8245, 1:1000, Abcam, UK) was adopted to normalize loading.

The primary antibodies RUNX2 (ab76956, 1:1000), Osterix (OSX) (ab229258, 1:500), Collagen I (COL-1) (ab34710, 1:2000), Osteocalcin (OCN) (ab93876, 1:1000), bax (ab32503, 1:2000), bcl-2 (ab32124, 1:1000), caspase-3 (ab32351, 1:5000), cleaved caspase-3 (ab32042, 1:500), and SOX11 (ab229185, 1:1000) were purchased from Abcam (Cambridge, UK).

### qRT-PCR

The total RNA was extracted from AFs using Trizol reagent (Takara, Dalian, China) and reversely transcribed into cDNA by a reverse transcription kit (DBI, USA) per the manufacturer’s protocols. QRT−PCR analysis was implemented using the ABI 7300 real−time PCR system. GAPDH and U6 were used for mRNA and miRNA normalization, respectively, and the 2^−ΔΔCt^ formula was used to measure the relative expression. The primer sequences (Table 2) were designed by Sangon Biotech (Shanghai, China).

**Table 2:** PCR primer sequences used in this study.

GAS5	Forward (5′–3′)	TGGATGACTTGCTTGGGTAAG
	Reverse (5′–3′)	TAACAGGTCTGCCTGCATTT
SOX11	Forward (5′–3′)	AGCAAGAAATGCGGCAAGC
	Reverse (5′–3′)	ATCCAGAAACACGCACTTGAC
miR-221-3p	Forward (5′–3′)	GCTACATTGTCTGCTGGGTGCTACATTGT
	Reverse (5′–3′)	CAGTTTTTTTTTTTTTTTGAAACCA
U6	Forward (5′–3′)	CGCTTCACGAATTTGCGTGTCAT
	Reverse (5′–3′)	GCTTCGGCAGCACATATACTAAAAT
GAPDH	Forward (5′–3′)	GGAGCGAGATCCCTCCAAAAT
	Reverse (5′–3′)	GGCTGTTGTCATACTTCTCATGG

### Bioinformatics analysis

GSE56081 (https://www.ncbi.nlm.nih.gov/geo/query/acc.cgi?acc=GSE56081) [39], a dataset containing the lncRNA and mRNA expression profiling of five normal and five degenerative disks, was downloaded to identify the upregulated lncRNA and mRNA in degenerative disks. The potential target lncRNA/mRNA of miR-221-3p and the corresponding binding sites were predicted using ENCORI (https://starbase.sysu.edu.cn/index.php) [40]. The target lncRNA/mRNA was obtained by intersecting the identified upregulated lncRNA/mRNA from GSE56081 and the potential target lncRNA/mRNA of miR-221-3p.

### Luciferase reporter assay

For dual-luciferase analysis, miR-221-3p mimics or NC mimics were co-transfected with SOX11-MUT, SOX11-WT, lncRNA GAS5-MUT, or lncRNA GAS5-WT into AF cells by the manufacturer’s protocols. The Dual-Luciferase Reporter Assay System (Promega, Madison, WI, USA) was then employed to measure the luciferase activity.

### RNA immunoprecipitation

RNA immunoprecipitation (RIP) was analyzed using the Magna RIP RNA-binding protein immunoprecipitation kit (Millipore, Darmstadt, Germany) through Anti-AGO2 (#03-110, Germany). QRT-PCR was adopted to measure the RNA-bound complexes, and isotype control was conducted using anti-IgG.

### Statistical analysis

Statistical analysis was performed using GraphPad Prism 7 (GraphPad, Boston, MA, USA). Students’ *t*-tests were employed to analyze the differences between the two groups. In addition, the differences of multiple groups were evaluated by one-way analysis of variance. Values are presented as mean ± SEM (standard error of the mean). A *P*-value of < 0.05 was considered statistical significance.

## Supplementary Materials

Supplementary Figures

Supplementary Tables 1 and 5

Supplementary Tables 2-4
