# Supplementary material for: GAS5, a long noncoding RNA, contributes to annulus fibroblast osteogenic differentiation and apoptosis in intervertebral disk degeneration via the miR-221-3p/SOX11 axis
Source: Aging (Albany NY). 2024 Feb 23;16(4):3896–914. doi: 10.18632/aging.205567 (PMC10929823; doi:10.18632/aging.205567)
Supplement: Supplementary Figures [file aging-16-205567-s001.pdf]

SUPPLEMENTARY FIGURES

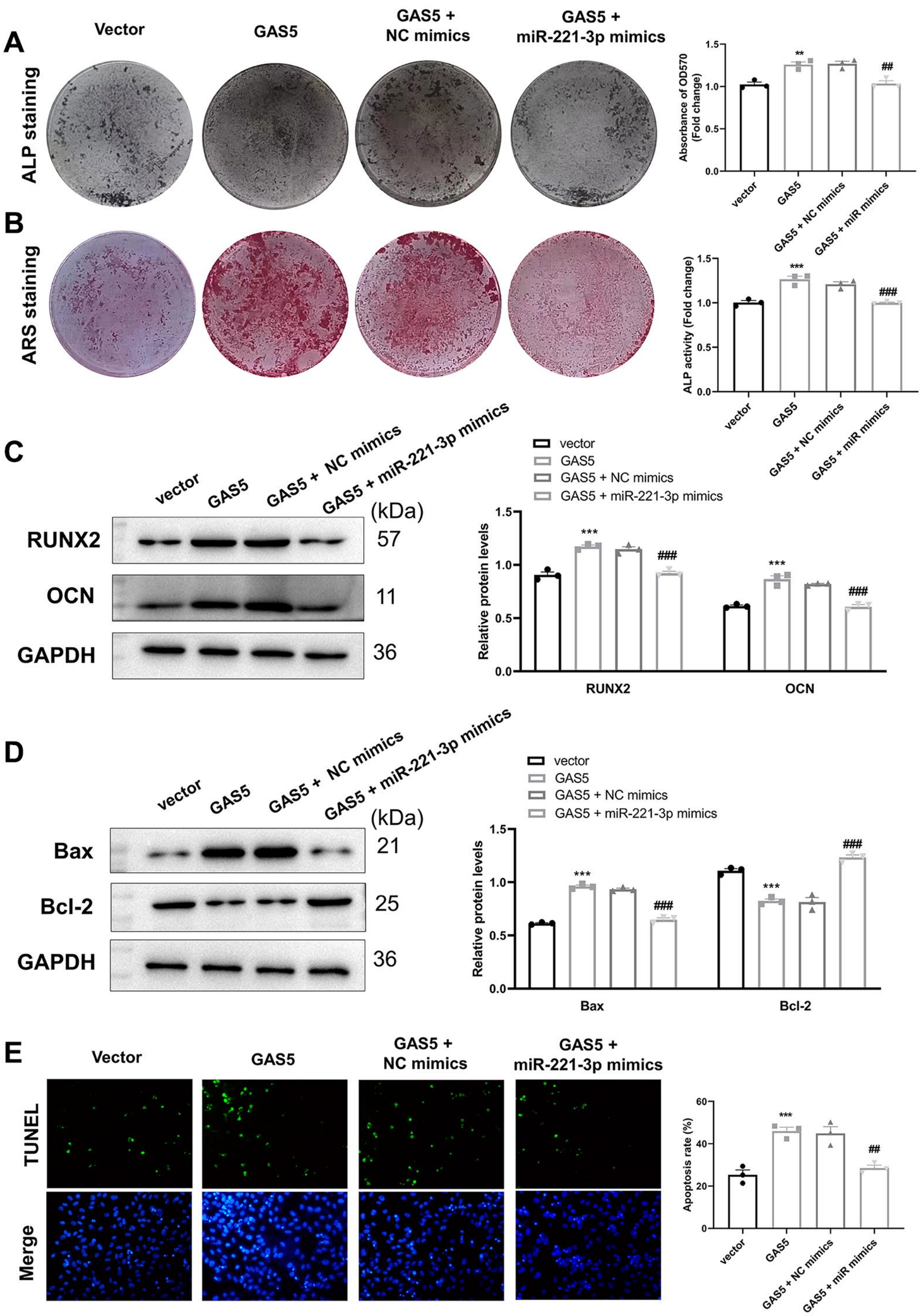

Supplementary Figure 1. The role of GAS5 in the apoptosis and osteogenic differentiation of AFs was mediated by miR-221-3p. AFs were transfected with or without the GAS5 overexpression vector alone or with the NC or miR-221-3p mimics. (**A**) ALP and (**B**) ARS staining assays were used to evaluate the osteogenic differentiation level (n = 3). (**C**) The osteogenic differentiation-related markers were detected using western blotting (n = 3). (**D**) The apoptosis-related protein levels were measured using western blotting (n = 3). (**E**) TUNEL staining assay was employed to evaluate the apoptosis of AFs (n = 3). ($^{**}p < 0.01$ and $^{***}p < 0.001$, vs. the vector group; $^{\#\#}p < 0.01$ and $^{\#\#\#}p < 0.001$, vs. the GAS5 group).

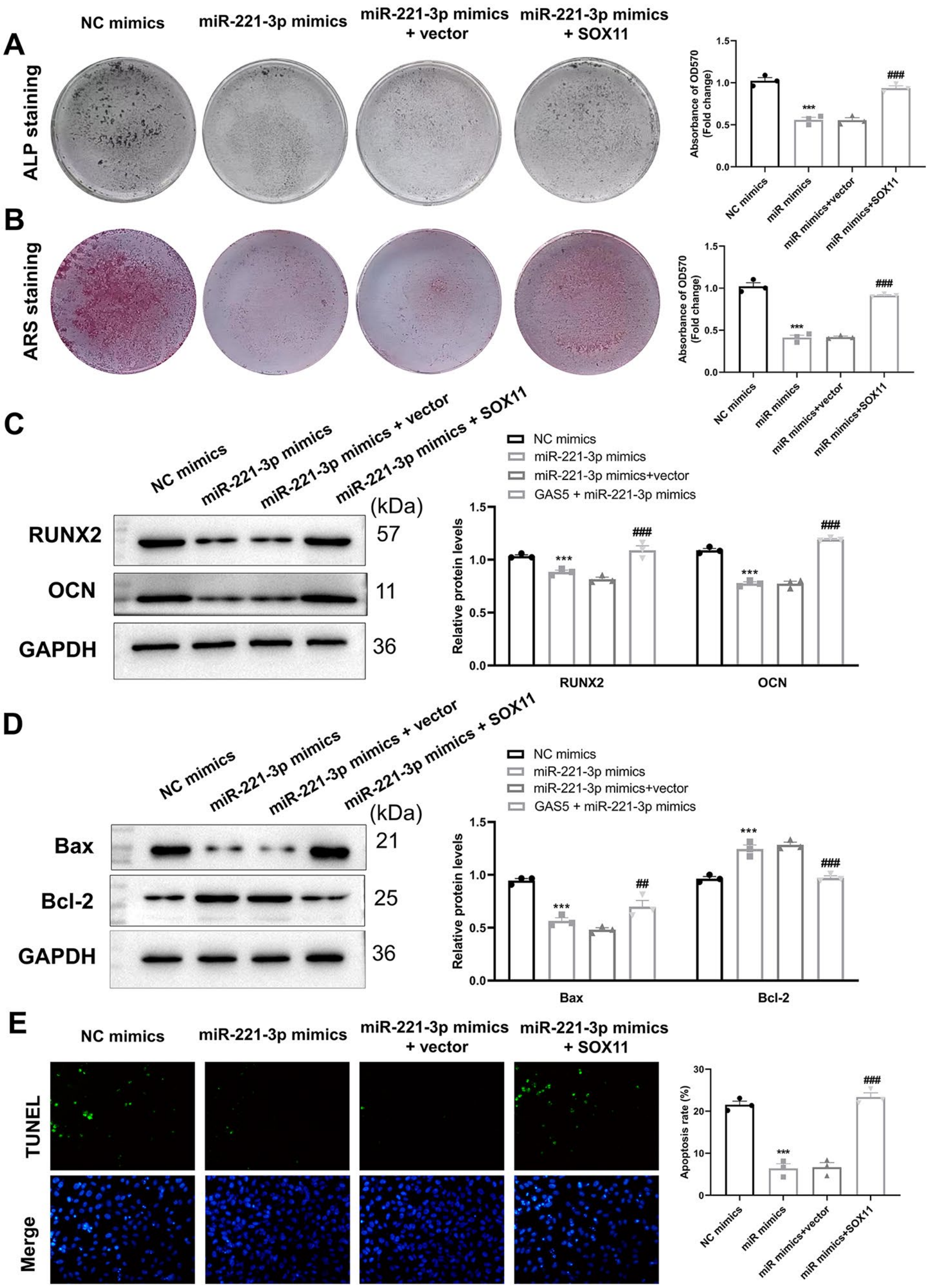

Supplementary Figure 2. SOX11 mediated the role of miR-221-3p in the apoptosis and osteogenic differentiation of Afs. AFs were transfected with or without miR-221-3p mimics alone or with the empty or SOX11 overexpression vector. (**A**) ALP and (**B**) ARS staining assays were used to evaluate the osteogenic differentiation level (*n* = 3). (**C**) The osteogenic differentiation-related markers were detected using western blotting (*n* = 3). (**D**) The apoptosis-related protein levels were measured using western blotting (*n* = 3). (**E**) TUNEL staining assay was employed to evaluate the apoptosis of AFs (*n* = 3). ($^{***}p < 0.001$, vs. the NC mimics group; $^{\#\#}p < 0.01$ and $^{\#\#\#}p < 0.001$, vs. the miR-221-3p mimics group).
