# Supplementary material for: GAS5, a long noncoding RNA, contributes to annulus fibroblast osteogenic differentiation and apoptosis in intervertebral disk degeneration via the miR-221-3p/SOX11 axis
Source: Aging (Albany NY). 2024 Feb 23;16(4):3896–914. doi: 10.18632/aging.205567 (PMC10929823; doi:10.18632/aging.205567)
Supplement: Supplementary Tables 1 and 5 [file aging-16-205567-s002.pdf]

## SUPPLEMENTARY TABLES

**Supplementary Table 1. The upregulated lncRNA in IVDD based on GSE56081.**

| Gene symbol | Regulation direction | Fold change | *p*-value | FDR |
|---|---|---|---|---|
| C14orf132 | up | 25.315744 | 0.0000575 | 0.002230592 |
| FLJ44054 | up | 12.046523 | 0.0000529 | 0.002158638 |
| TMEM41B | up | 10.290109 | 0.000803 | 0.009374719 |
| H19 | up | 9.438205 | 0.000526 | 0.007148484 |
| MDM4 | up | 9.3215885 | 0.002827955 | 0.019875953 |
| PMS2L3 | up | 7.177083 | 0.00314208 | 0.021223195 |
| MALAT1 | up | 6.855616 | 0.004708168 | 0.027079232 |
| C10orf75 | up | 6.6556325 | 0.000394 | 0.005983249 |
| SNHG8 | up | 6.5955076 | 0.00083 | 0.009555447 |
| RPL10 | up | 6.124247 | 0.00000973 | 0.001106426 |
| LOC728855 | up | 6.1202617 | 0.002670312 | 0.019144418 |
| MGC23270 | up | 6.11878 | 0.00000957 | 0.001106426 |
| COX11 | up | 6.0228524 | 0.000306 | 0.005141456 |
| RNF126P1 | up | 5.7088127 | 0.000487 | 0.006878821 |
| LOC96610 | up | 5.559347 | 0.000549 | 0.007337372 |
| SBF1P1 | up | 5.491599 | 0.00346884 | 0.022488918 |
| NCRNA00188 | up | 5.452553 | 0.00140898 | 0.012828844 |
| GAS5 | up | 5.403014 | 0.000681 | 0.008418643 |
| HNRPA1L-2 | up | 5.0491734 | 0.000142 | 0.003403639 |
| SCARNA23 | up | 4.9850054 | 0.000103 | 0.002904538 |
| CATSPER2P1 | up | 4.806248 | 0.00325512 | 0.021722205 |
| LOC404266 | up | 4.666092 | 0.001056403 | 0.010741591 |
| RGL2 | up | 4.5703983 | 0.000404 | 0.006064587 |
| LOC645166 | up | 4.4355288 | 0.001016234 | 0.010493009 |
| SNHG6 | up | 4.274251 | 0.000386 | 0.00590595 |
| LOC441601 | up | 3.9893003 | 0.0000628 | 0.002335666 |
| CIRBP | up | 3.9523647 | 0.000481 | 0.006829181 |
| RPL21P28 | up | 3.9490988 | 0.000911 | 0.010032637 |
| LOC285696 | up | 3.5922666 | 0.001126515 | 0.011189451 |
| IFITM4P | up | 3.540142 | 0.0000692 | 0.002394151 |
| RPL23AP7 | up | 3.4901786 | 0.002118995 | 0.016462892 |
| GBAP1 | up | 3.2741895 | 0.000912 | 0.010032637 |
| LOC647979 | up | 3.1333585 | 0.000582 | 0.007589122 |
| LOC649395 | up | 2.7442327 | 0.0000396 | 0.001908888 |

**Supplementary Table 5. The potential target mRNAs of miR-221-3p that up-regulated in IVDD.**

| |
|---|
| SLC7A6 |
| ADAM12 |
| EIF1 |
| OTUD4 |
| CAMTA1 |
| TFR2 |
| ZFP36 |
| HNRNPA0 |
| KLHL21 |
| PPFIBP1 |
| MGP |
| HMBOX1 |
| BSDC1 |
| SLC5A3 |
| SOCS3 |
| KLF9 |
| LFNG |
| GPM6A |
| HMGCR |
| PFKFB2 |
| MAPK1 |
| ATF2 |
| DDX17 |
| PDE4DIP |
| GAS1 |
| BNIP3L |
| TOLLIP |
| KCNMB4 |
| DNAJB14 |
| CYP1B1 |
| ZNF22 |
| MEF2D |
| SLC25A37 |
| CHSY1 |
| PRDX6 |
| DICER1 |
| MYO1C |
| GREB1 |
| LSM14A |
| TIPARP |
| BCL2L2 |
| LTBP2 |
| TRAPPC10 |
| ATF4 |
| UBE2G2 |
| BRD9 |

| |
|---|
| BTBD3 |
| GIT1 |
| KLF8 |
| FOS |
| CEBPG |
| SCARB2 |
| H1FX |
| KLHDC10 |
| CYTH3 |
| MLEC |
| MEST |
| CHD5 |
| CCND2 |
| SOX11 |
| SPARC |
| KCTD12 |
| IGFBP4 |
| LANCL1 |
